# Genetic associations with suicide attempt severity and genetic overlap with major depression

**DOI:** 10.1038/s41398-018-0340-2

**Published:** 2019-01-17

**Authors:** Daniel F. Levey, Renato Polimanti, Zhongshan Cheng, Hang Zhou, Yaira Z. Nuñez, Sonia Jain, Feng He, Xiaoying Sun, Robert J. Ursano, Ronald C. Kessler, Jordan W. Smoller, Murray B. Stein, Henry R. Kranzler, Joel Gelernter

**Affiliations:** 1 Division of Human Genetics, Department of Psychiatry, Yale University School of Medicine, New Haven, CT USA; 2 Department of Psychiatry, Veterans Affairs Connecticut Healthcare Center, West Haven, CT USA; 3 Department of Family Medicine and Public Health, University of California San Diego, La Jolla, CA USA; 4 Uniformed Services University of the Health Sciences, Bethesda, MD USA; 5 Department of Health Care Policy, Harvard Medical School, Boston, MA USA; 6 Department of Psychiatry, Massachusetts General Hospital, Harvard Medical School, Boston, MA USA; 7 Psychiatric and Neurodevelopmental Genetics Unit, Center for Genomic Medicine, Massachusetts General Hospital, Boston, MA USA; 8 Stanley Center for Psychiatric Research, Broad Institute of MIT and Harvard, Cambridge, MA USA; 9 Department of Psychiatry, University of California San Diego, La Jolla, CA USA; 10 VA San Diego Healthcare System, San Diego, CA USA; 11 Department of Psychiatry, University of Pennsylvania Perelman School of Medicine, Philadelphia, PA USA; 12 Veterans Integrated Service Network 4 Mental Illness Research, Education and Clinical Center, Crescenz Veterans Affairs Medical Center, Philadelphia, PA USA; 13 Department of Genetics, Yale University School of Medicine, New Haven, CT USA; 14 Department of Neuroscience, Yale University School of Medicine, New Haven, CT USA

## Abstract

In 2015, ~800,000 people died by suicide worldwide. For every death by suicide there are as many as 25 suicide attempts, which can result in serious injury even when not fatal. Despite this large impact on morbidity and mortality, the genetic influences on suicide attempt are poorly understood. We performed a genome-wide association study (GWAS) of severity of suicide attempts to investigate genetic influences. A discovery GWAS was performed in Yale-Penn sample cohorts of European Americans (EAs, *n* = 2,439) and African Americans (AAs, *n* = 3,881). We found one genome-wide significant (GWS) signal in EAs near the gene *LDHB* (rs1677091, *p* = 1.07 × 10^−8^) and three GWS associations in AAs: *ARNTL2* on chromosome 12 (rs683813, *p* = 2.07 × 10^−8^), *FAH* on chromosome 15 (rs72740082, *p* = 2.36 × 10^−8^), and on chromosome 18 (rs11876255, *p* = 4.61 × 10^−8^) in the Yale-Penn discovery sample. We conducted a limited replication analysis in the completely independent Army-STARRS cohorts. rs1677091 replicated in Latinos (LAT, *p* = 6.52 × 10^−3^). A variant in LD with FAH rs72740082 (rs72740088; *r*^2^ = 0.68) was replicated in AAs (STARRS AA *p* = 5.23 × 10^−3^; AA meta, 1.51 × 10^−9^). When combined for a trans-population meta-analysis, the final sample size included *n* = 20,153 individuals. Finally, we found significant genetic overlap with major depressive disorder (MDD) using polygenic risk scores from a large GWAS (*r*^2^ = 0.007, *p* = 6.42 × 10^−5^). To our knowledge, this is the first GWAS of suicide attempt severity. We identified GWS associations near genes involved in anaerobic energy production (*LDHB*), circadian clock regulation (*ARNTL2*), and catabolism of tyrosine (*FAH*). These findings provide evidence of genetic risk factors for suicide attempt severity, providing new information regarding the molecular mechanisms involved.

## Introduction

Suicide is a conscious act made with the intent to end one’s own life and is thus uniquely human, although environmental, genetic, and neuroanatomical dimensions underlying these behaviors may be shared with other species^1^. Suicide is a challenging and complex public health problem; 44,965 people died by suicide in the United States in 2016, the most recent year for which statistics are available (https://webappa.cdc.gov/sasweb/ncipc/leadcause.html), making it the tenth leading cause of death and the second leading cause of death among people age 10–45 in the United States.

To die by suicide, one must attempt it, and this act is preceded by suicidal ideation (although in some cases the ideation is merely a momentary impulse which precedes an attempt). Many people who experience suicidal ideation never attempt suicide, and most who attempt suicide do not die from that attempt. Many cases when individuals do not progress from each of the preceding steps—from ideation to an attempt towards the final act of death by suicide—may be distinct biologically from those that do. A recent application of polygenic risk scores that aimed to predict ideation from attempt and vice versa failed to provide evidence of overlapping genetic factors in these phenotypes but was underpowered^2^. We would predict high heterogeneity for suicidal ideation because it is the broadest expression of suicidal behavior and less likely to progress to death by suicide than suicide attempt. Ideation arises from a broad variety of causes and may be considered prodromal for suicide attempt and death by suicide, but suicidal ideation as a category contains both individuals who will eventually attempt suicide and many who will never progress farther along this behavioral spectrum.

Recent reviews of genetic associations with suicidal behavior have highlighted the difficulty of identifying significant common variants^3,4^. To our knowledge there have not been any genome-wide significant (GWS) associations reported for suicidal ideation. This may reflect the heterogeneity expected for suicidal ideation and consequent need for large samples to detect common risk variants. There has been greater success in studies of suicide attempts, with studies identifying associated risk variants at the GWS threshold in subjects with bipolar disorder^5^, GWS in a non-clinical population of US soldiers^6^ and a recent case-cohort study from Denmark^7^.

We focused on a quantitative measure of suicide attempts. We excluded from analyses subjects with suicidal ideation but no attempts, because of the potential to misclassify them phenotypically: we did not classify them with the control group because a relatively high percentage of these subjects become attempters. In this way, we aimed to reduce sample heterogeneity and capture degrees of severity within suicide attempters. While a previous GWAS examined a quantitative suicidality score^8^, the scale included both ideation and attempt on a single continuum. Other GWAS of suicidal behavior have used case-control designs, which may limit power by forcing individual participants into a binary categorization that may not reflect the true spectrum of severity in suicide attempt behavior^9^.

## Methods

### Participants

Participants for the discovery GWAS in this study were recruited from five sites in the eastern United States, for studies of the genetics of drug or alcohol dependence - the Yale-Penn study^10–13^. All participants were interviewed using the Semi-Structured Assessment for Drug Dependence and Alcoholism (SSADDA)^14^, which contains several items relevant to suicidal behavior, as discussed below. Participants provided written informed consent and the study was approved by the institutional review board at each participating site (Yale Human Research Protection Program, University of Pennsylvania Institutional Review Board, University of Connecticut Human Subjects Protection Program, Medical University of South Carolina Institutional Review Board for Human Research, and the McLean Hospital Institutional Review Board). For replication, we used the Army Study to Assess Risk and Resilience in Servicemembers (Army-STARRS): New Soldier Study (NSS) and Pre-/Post-Deployment Study (PPDS). NSS was carried out among new soldiers beginning basic training at three Army Installations between April 2011 and November 2012, and has also been described previously^6^. PPDS data were collected from US Army soldiers within ~6 weeks prior to a deployment to a theater of combat operations. The replication cohort was younger and more male than the Yale-Penn cohort, with a mean age of ~25 and ~3% female subjects.

### Suicide Attempt Scale

A quantitative phenotype for suicide attempt severity was derived from the SSADDA data (Table 1). Participants who reported suicidal ideation with no suicide attempts were excluded from the study. Participants were scored 0 if no attempt or suicidal ideation was reported, 1 if they reported at least one attempt, 2 if they had received treatment for a suicide attempt, 3 if they had a medical hospitalization due to a suicide attempt, and a 4 if the method of suicide attempt was classified as violent based on the Asberg criteria of methods other than overdose^15^. Phenotype scores thus ranged from 0 to 4. Of the 8318 subjects with both genotype and phenotype data available, 1,998 subjects with suicidal ideation but without a suicide attempt were excluded, leaving 6,320 individuals (1,131 affected) for the analysis of suicide attempt severity, including 2,439 European American (EAs) subjects (488 affected) and 3,881 African American (AAs) subjects (643 affected).

**Table 1:** Phenotype Definition, Scoring, Demographics, and Comorbidty

Cohort		All Participants	Participants with severity score				
			0	1	2	3	4
			No Suicidal Ideation/thoughts (Have you ever thought about killing yourself?)	Suicide Attempt (Have you ever tried to kill yourself?)	Received treatment for suicide attempt (Did you require medical treatment after you tried to kill yourself?)	Medical hospitalization due to suicide attempt (Were you admitted to a hospital after the attempt for medical reasons?)	Violent Method (Knife/Cutting, Hanging, Jump from height, Firearm, Poison, Vehicle (walked into traffic), Crash car, Fire, Electrocution)
Yale Penn 1 EA	n	1225	894	52	53	78	148
	Mean Age	37.91	37.79	38.96	38.68	37.63	38.11
	%Female	41%	38%	52%	60%	62%	43%
	%MDD	15%	12%	27%	28%	35%	20%
	%BP	6%	3%	10%	11%	17%	18%
	%SZ	0%	0%	0%	0%	0%	1%
	%SUD	97%	96%	100%	100%	100%	100%
Yale Penn 2 EA	n	1214	1057	40	32	30	55
	Mean Age	39.17	39.09	41.03	38.63	34.6	42.04
	%Female	42%	39%	58%	72%	47%	53%
	%MDD	14%	10%	45%	44%	43%	35%
	%BP	4%	3%	13%	13%	20%	4%
	%SZ	0%	0%	0%	0%	0%	2%
	%SUD	76%	73%	98%	94%	100%	100%
Yale Penn 1 AA	n	2530	2080	114	100	83	153
	Mean Age	41.13	41.42	39.98	40.29	41	38.65
	%Female	48%	46%	68%	71%	63%	49%
	%MDD	11%	8%	22%	18%	39%	24%
	%BP	3%	2%	9%	8%	12%	7%
	%SZ	0%	0%	0%	1%	0%	1%
	%SUD	85%	82%	94%	98%	98%	97%
Yale Penn 2 AA	n	1351	1158	45	42	29	77
	Mean Age	40.89	41.01	40.53	39.88	41.66	39.49
	%Female	42%	40%	64%	55%	66%	42%
	%MDD	9%	6%	27%	21%	38%	23%
	%BP	2%	2%	11%	5%	0%	4%
	%SZ	0%	0%	0%	0%	0%	1%
	%SUD	69%	64%	91%	98%	93%	97%

### Genotyping, imputation, and quality control

For Discovery, we included two different sets of identically ascertained subjects who were genotyped on different platforms (Table 2). Yale-Penn 1, collected earlier, was genotyped using the HumanOmni-Quad v1.0 array (Illumina) containing 988,306 autosomal single nucleotide polymorphism (SNP) markers. Yale-Penn 2 was genotyped on the HumanCore Exome array (Illumina) containing 550,601 SNPs. The methods used for imputation and quality control have been reported previously^13^. Individuals and SNPs with call rates <98% were removed. Only imputed SNPs with an accuracy greater than 0.8 were retained, and all SNPs with a Hardy-Weinberg equilibrium P < 10^−5^ were removed. SNPs with MAF < 1% were removed. Subject population was defined based on two ancestry groups, European American (EA) and African American (AA), assigned using 1000 genome phase 3 for EUR and AFR as reference.

**Table 2:** Replication of top SNPs in STARRS samples

African Americans			Yale-Penn AA		STARRS			
rsid	Allele1	Allele2	Yale-Penn P-value	Zscore	AA P-value	AA Beta		
rs72740088	t	c	7.49 × 10^−8^	−5.379	5.23 × 10^−3^	−0.068		
rs61520094	t	c	8.14 × 10^−7^	4.932	0.228	0.0164		
rs10867557	a	t	4.73 × 10^−7^	−5.037	0.472	0.0116		
EAs and Latinos			Yale-Penn EA		STARRS			
rsid	Allele1	Allele2	Yale-Penn P-value	Zscore	EA P-value	EA Beta	Latino P-value	Latino Beta
rs1677091	a	c	1.07 × 10^−8^	5.719	0.7434	−0.0014	6.52 × 10^−3^	0.0199
rs860447	t	c	6.10 × 10^−7^	4.988	0.6043	0.0022	6.5 × 10^−3^	0.02

For the Army-STARRS replication sample, genotyping methods have also been previously described^6^. NSS1 and PPDS samples were genotyped using the Illumina OmniExpress + Exome array, while the NSS2 sample was genotyped on the Illumina PsychChip. Imputation was done using a reference multi-ethnic panel from the 1000 Genomes Project. Population assignment for the Army-STARRS sample using 1000 genome phase 3 data for CEU (Utah residents with Northern and Western European ancestry), YRI (Yoruba in Ibadan, Nigeria), and MXL (Mexican ancestry in Los Angeles, California) as reference yielded three ancestry groups: European American (EA), African American (AA), and Latino (LAT) assigned, respectively. Assignment to LAT in Army-STARRS and not in Yale-Penn reflects how these two studies have handled population group assignment previously. In Yale-Penn, Latino subjects are classified as EA or AA if their ancestry corresponds to one of these groups, or excluded as population outliers if they are not mostly EA or AA. This decision was taken early in the Yale-Penn study because Latinos in the Eastern US, where Yale-Penn is based, can mostly be assigned in this way: the Latino population is defined based on a shared language, and not based on a particular shared ancestry, in contrast to EA and AA.

### Statistical analysis

Analysis was performed independently in 4 different cohorts for suicide attempt severity: Yale-Penn 1 and Yale-Penn 2 AAs; and Yale-Penn 1 and Yale-Penn 2 EAs. Some of the subjects recruited from this sample were originally recruited for family linkage studies and are members of small nuclear families^16,17^. To control for these related subjects, PLINK v1.9 ^18^ was used to prune SNPs in LD and Genome-Wide Efficient Mixed Model Association (GEMMA)^19^ was used to generate a relatedness matrix. GEMMA was then used on the unpruned data to perform a Wald test while adjusting for age, sex, the first 10 principal components, and the degree of relatedness between subjects. METAL was used to perform meta-analyses between Yale-Penn 1 and Yale-Penn 2 cohorts from the resulting summary statistics for EAs and AAs taken separately. These two populations were then meta-analyzed together. A final trans-population meta-analysis was also performed for all 4 cohorts. GCTA was used on unrelated subjects to identify the SNP-based heritability in the Yale Penn EA and AA cohorts.

### Polygenic risk scores

PRS analysis was conducted using PRSice v 1.25^20^. Summary statistics from the Psychiatric Genomics Consortium (PGC) and 23andMe GWAS of MDD^21^ were used to calculate the PRS. Genetic overlap between MDD and suicide attempt severity was tested in the combined EA Yale-Penn cohorts. Related individuals were removed from the Yale-Penn sample, leaving 2292 individuals (of the original 2439) for PRS testing.

### Gene-based test

Summary statistics from the meta-analyses were loaded into Functional Mapping and Annotation of Genome-Wide Association Studies (FUMA GWAS) to test for gene-based associations^22^. Input SNPs were mapped to 17,927 protein coding genes. The GWS threshold for the gene-based test was determined to be *p* = 0.05/17,927 = 2.79 × 10^−6^.

### Conditional Analysis

Conditional analyses were used to determine how many independent signals were present among the SNPs with at least suggestive association evidence (*p* ≤ 1 × 10^−6^, Supplementary Tables 1 and 2). GWAS was rerun for each of the suggestive signals in EAs and AAs, conditioned on the top SNP from each region as covariates. None of the other regional signals were significant following the conditional analysis. Following conditional analyses, there were 15 independent regions (5 in EAs, 10 in AAs) where we sought to test for replication. Only three of these independent regions were available in Army-STARRS for testing in EAs and AAs, respectively.

### Replication in Army-STARRS samples

A quantitative trait was constructed similar to the one applied in Yale-Penn, except categories 1–3 were combined so that a 0 meant no suicidal ideation or attempt, 1 meant any suicide attempt had occurred, and 2 meant that a violent method was used. This was done because there were few subjects classified as levels 2 and 3 based on the information available. (Supplementary Table S4). The scores thus ranged from 0 to 2. The 826 individuals in the AAs PPDS cohort were excluded due to inflation, leaving 13,833 total subjects in the replication cohorts, with 9,382 EAs (400 affected), 1488 AAs (83 affected), and 2963 LATs (108 affected)^6^.

GWA analyses were conducted on the suggestive SNPs from the Yale-Penn cohort in the Army-STARS cohorts using PLINK v1.90^18^ as previously described^6^, except that a linear regression model was used. Sex, age, and the first 10 PCs within ancestry were included as covariates. Conditional analyses were conducted (see above) to identify how many independent signals were present from each of the Yale-Penn EA and AA cohorts. Of the 113 top suggestive SNPs in the Yale-Penn EAs data (see below), 98 were available for the STARRS EA and LAT analysis. Those mapped to three distinct associated regions in EAs that were GWS or for which there was suggestive evidence *p* ≤ 1 × 10^−6^ in the Yale-Penn meta-analysis (see below) and were tested in the STARRS EA and LAT cohorts. In the Yale-Penn sample, Latino subjects are mostly allocated genetically to the EA or AA groups, and accordingly, we also tested the top EA SNPs in the STARRS LAT sample. For AAs, only 4 of the 30 suggestive SNPs from the Yale-Penn analysis were available and tested in the STARRS AA cohorts. Based on the conditional analysis, only 3 independent signals were represented from each of the EA and AA cohorts. We included all of these suggestive SNPs to increase the likelihood that we would find acceptable proxies for the lead SNPs among available SNPs in the Army-STARRS GWAS. For EAs and LAT there were 98 SNPs available representing 3 independent signals. For AAs there were only 4 available SNPs for testing, corresponding to 3 independent signals. Following adjustment for multiple comparisons the corrected α for replication was 0.05/3 = 0.0166 in each of the cohorts.

## Results

### GWAS of Suicide Attempt Severity

**Fig. 1:**
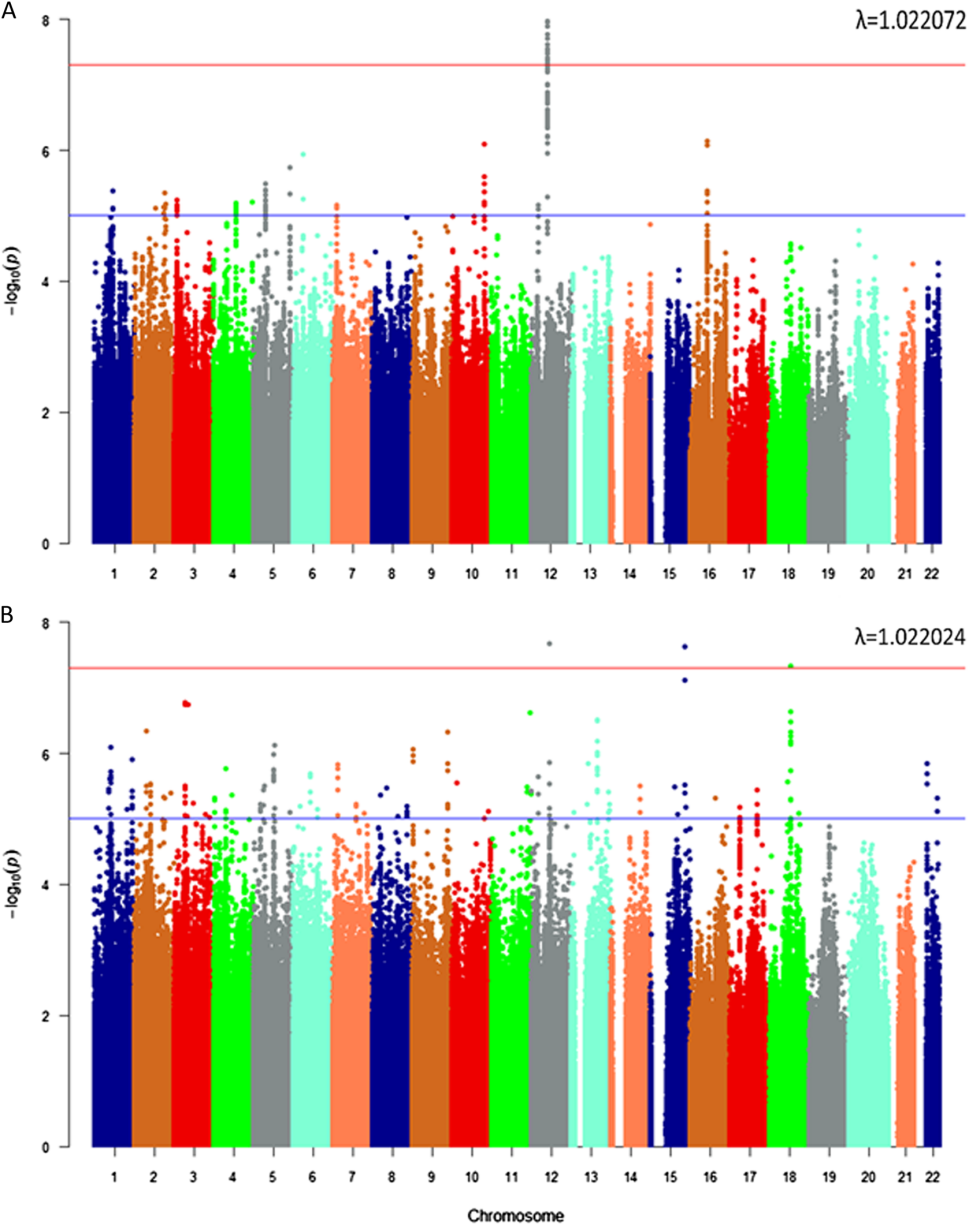
Manhattan plot. **a** European Americans with Lambda Value. **b** African Americans with Lambda Value

**Fig. 2:**
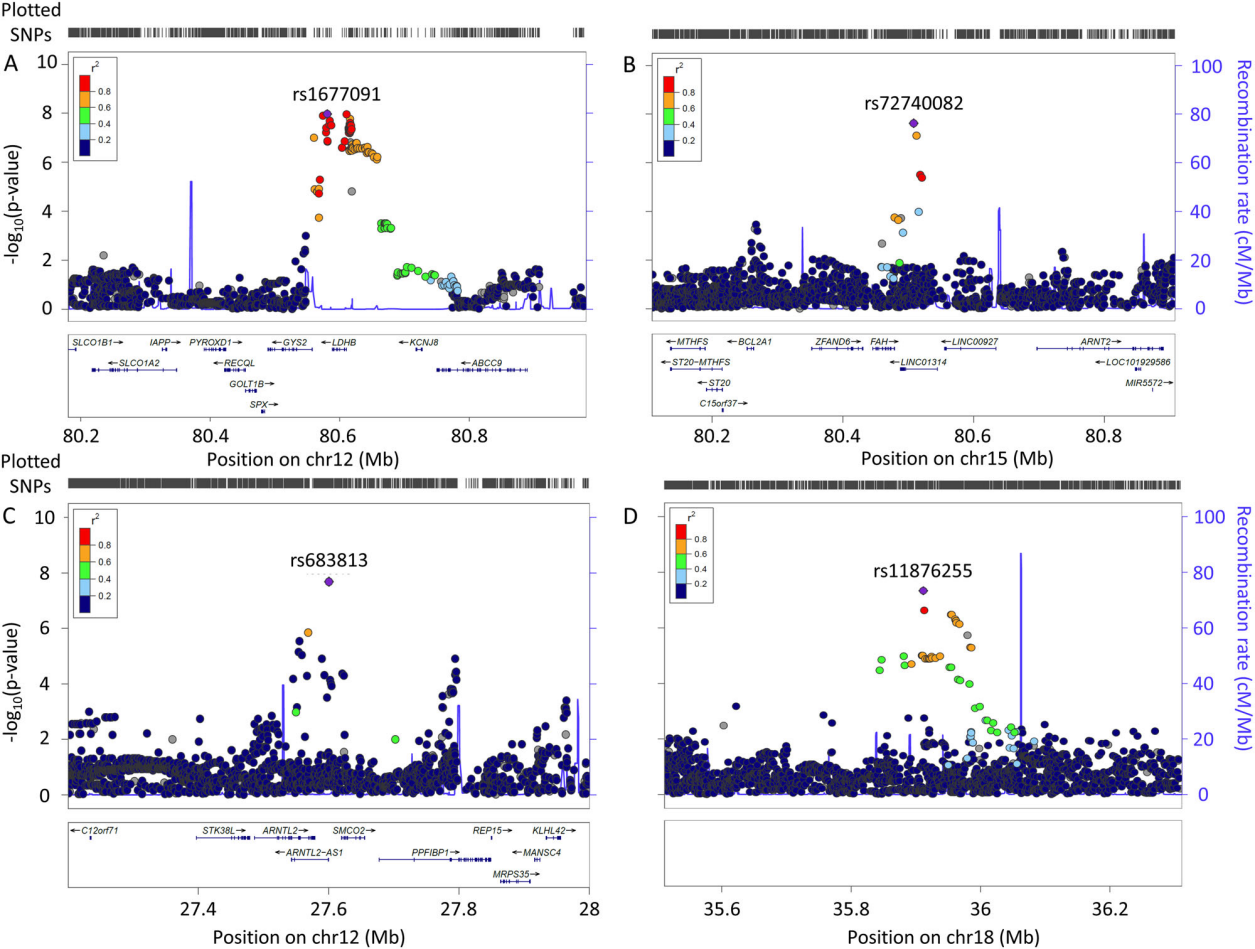
Regional Manhattan plots of top findings for suicide attempt severity. Top findings in European Americans on chromosome 12 (**a**). Top findings in African Americans on chromosome 15 near FAH (**b**), on chromosome 12 near ARNTL2 (**c**), and in the intergenic region on chromosome 18 (**d**)

There was one signal in the EAs (meta-analysis, Fig. 1a) in the Yale-Penn discovery cohort, with 34 GWS SNPs in high LD over 44 kb. These SNPs mapped in and around the “Lactate Dehydrogenase B” (*LDHB*) gene (Fig. 2a, Supplementary Table S2). The lead SNP (rs1677091, MAF = 0.30, *p* = 1.07 × 10^−8^) was directly genotyped in both cohorts.

Three independent GWS associations were found in the AAs (meta-analysis, Fig. 1b). The first association (rs683813, MAF = 0.04, *p* = 2.07 × 10^−8^) was 13 kb upstream of the “Aryl Hydrocarbon Receptor Nuclear Translocator Like 2 antisense RNA 1” (*ARNTL2-AS1*) gene (Fig. 2b). A second association (rs72740082, MAF = 0.027, *p* = 2.36 × 10^−8^) was on chromosome 15 within “Cortexin Domain Containing 1” (*CTXND1*), “Long Intergenic Non-Protein Coding RNA 1314” (*LINC01314*), and adjacent to “Fumarylacetoacetate Hydrolase” (*FAH*). (Fig. 2c, Supplementary Table S3). A third association (rs11876255, MAF = 0.03, *p* = 4.61 × 10^−8^) was in an intergenic region of chromosome 18 (Fig. 2d). Considering the low MAFs observed for the associated variants and the quantitative phenotype, we tested whether the finding was likely to be correct in the context of the distribution of the phenotype. Suicide attempts are relatively rare in the population; only ~20% of our cohort had a suicide attempt score > 0. We used a permutation analysis that assigned phenotypes randomly, while maintaining the same distribution to re-run the meta-analysis. After 1000 permutations for each of the top findings we saw no evidence that the phenotype distribution biased our findings. (Supplementary Figure 1)

### Polygenic risk score

**Fig. 3:**
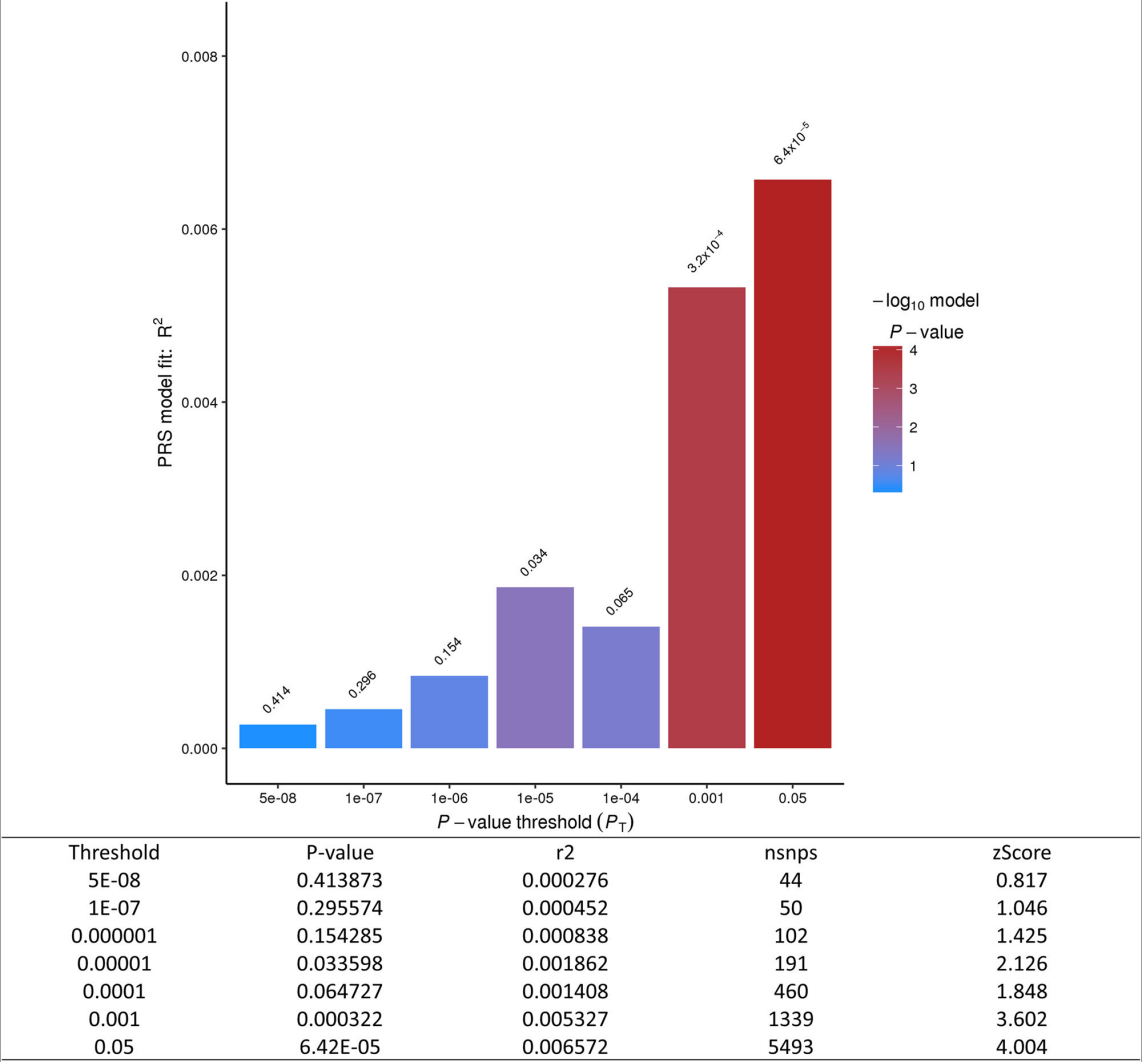
Polygenic Risk Score. Genetic overlap with MDD was tested by generating a polygenic risk score from a large PGC/23andMe GWAS and testing in the combined Yale-Penn EA cohort (*n* = 2292)

We tested genetic overlap using MDD polygenic risk scores (PRS)^21^ using PRSice v1.25^20^. We found significant genetic overlap: the PGC-MDD PRS can explain up to 0.7% of the variance of suicide attempt severity in Yale-Penn (z = 4.00; *p* = 6.42 × 10^5^; Fig. 3).

### Replication in Army-STARRS sample

A total of 98 SNPs from the Yale-Penn discovery analysis were looked up in the STARRS EA and LAT samples. None (lead replication SNP: rs860447, MAF = 0.26, *p* = 0.60, Yale-Penn/STARRS EA-EA meta *p* = 6.4 × 10^−3^) replicated in the STARRS EA data, but 34 of the top 98 EA SNPs were nominally significant in the STARRS LAT samples, and, remarkably, all 98 had effect direction consistent with what we observed in Yale-Penn EAs; the lead SNP from Yale-Penn EAs was nominally significant in STARRS LATs (rs1677091, MAF = 0.28 *p* = 6.52 × 10^−3^) and improved in a meta-analysis of the Yale-Penn EA and STARRS LAT samples (meta *p* = 4.7 × 10^−9^). This could be attributable to Latino admixture in the Yale-Penn EAs; or to the fact that the individuals in the Army-STARRS sample were much younger and had traversed less of their lifetime risk period for suicide. Accordingly, suicide-related diagnoses in the Army STARRS sample are, on average, of earlier onset, and presumably represent a more severe phenotype. Regarding possible influence of LAT admixture in the Yale-Penn results, the MAF for rs1677091 in Yale-Penn (MAF = 0.30) is nearly identical to the 1000 genome phase 3 for EUR (MAF = 0.29), so stratification is not a good explanation.

Only four of the 30 top SNPs from the Yale-Penn meta-analysis of AAs were available for query in the STARRS AA data; one of these was nominally significant (rs72740088, MAF = 0.08 *p* = 5.22 × 10^−3^). When STARRS AA data were meta-analyzed with the Yale-Penn AA data, the statistical significance improved (meta *p* = 1.51 × 10^−9^).

This single top SNP was tested in a trans-populational meta-analysis of EAs, AAs, and LATs in all of the available STARRS cohorts. The overall meta of all 20,153 (1,728 affected) subjects in the Yale-Penn and STARRS cohorts for this SNP is suggestive (*p* = 7.84 × 10^−7^), possibly indicating heterogeneity.

### Association data for SNP alleles that are top findings for suicide, in substance use disorders

The Yale-Penn cohort was collected mostly for study of the genetics of substance use disorders (SUDs) and has previously been used to identify genetic associations for SUD traits. To evaluate whether the top findings for suicide are independent from those for SUDs, we looked up SNPs from previously published work using the Yale-Penn cohort for alcohol^10^, cocaine^12^, and cannabis^23^ dependence. One SNP was filtered prior to analysis in the alcohol GWAS due to MAF < 0.03 (rs72740082 MAF = 0.027), so we looked up the same surrogate SNP in high LD used in the Army-STARRS replication analysis (rs72740088, MAF = 0.08) above. None of the lead SNPs for suicide were even nominally significant in the SUD GWAS (Supplementary Table S6).

### Gene-based test and enrichment analysis of the Yale-Penn GWAS

As stated above, the significance cutoff for the gene-based test was set at *p* < 2.79 × 10^−6^. Gene-based testing identified a single GWS gene associated with suicide attempt in AAs at the “NMDA Receptor-Regulated 2” gene (*NARG2*, *p* = 2.64 × 10^−6^) (Supplementary Figure S2).

For EAs, 3 GWS genes were identified. These were *LDHB* (p = 6.57 × 10^−7^), which had numerous individual GWS SNPs as well, along with “PiggyBac Transposable Element Derived 5” (*PGBD5*, *p* = 2.41 × 10^−7^), and “Pleckstrin Homology-Like Domain, Family B, Member 2” (*PHLDB2*, *p* = 1.71 × 10^−6^) (Supplementary Figure S3).

MAGMA Gene-Set Analysis was carried out using the competitive model for each of the meta-analyses. For the trans-population meta-analysis of all 4 Yale-Penn cohorts the caspase pathway gene-set survived multiple testing corrections (*p* = 2.35 × 10^−7^).

## Discussion

We present the first quantitative trait GWAS of suicide attempts. We obtained novel findings that overlapped with the emerging biological model of suicidality. Our results from gene-based approaches support previously reported links to apoptosis, and additionally, GWS SNPs were identified in proximity to genes involved in the regulation of circadian rhythms, energy production, and catecholamine catabolism. Two design features—the use of a quantitative phenotype, and the exclusion of subjects with suicidal ideation but no history of suicide attempts—may have increased the statistical power to identify genetic associations in both available populations, AAs and EAs.

In constructing our scale from available data in the SSADDA, we did not assign differential severity weighting based on the number of suicide attempts for an individual because, while a previous suicide attempt is one of the largest risk factors for death by suicide, the number of past suicide attempts beyond the initial attempt does not necessarily reflect increased risk; the opposite may be true because individuals who attempt suicide attempt frequently without completing have self-evidently not selected highly lethal methods, so may have less desire to die and lower intent to harm themselves. While previous suicide attempts and self harm increase the risk of future suicide attempt or death, most suicide attempters do not go on to die by suicide^24–26^, Most deaths by suicide occur on the first known attempt^27,28^.

Classical work^29^ on the complexity of suicidal behavior and intent have examined quantitative scales for suicide, for example, the Suicidal Intent Scale (SIS), a 15 item questionnaire containing objective details on the nature of the suicide attempt (questions 1–8) and subjective self report of the attempter. Items from the SIS cluster into 6 factors that might better represent specific dimensions of suicidal behavior than scoring all items in one general scale^30^. The work of Beck informed modern scales such as the Columbia-Suicide Severity Rating Scale (C-SSRS) which seek to distinguish domains of suicidal ideation and behavior, which could have great value in the design of future prospective studies for domains of suicidal behavior^31^. While the SSADDA instrument predates the C-SSRS, our approach was informed by evidence that subtypes are present within suicidal behavior^32,33^

Our scale derived from the SSADDA resembles Beck’s Factor I, “attitudes toward attempt”. Accordingly, we scored severity based on the lethality of the attempt according to whether subjects required medical treatment or hospitalization following the attempt, or lethality of the method using available data from the SSADDA instrument.

Overall, SNP-based heritability within EAs was ascertained by GCTA on unrelated subjects in the EA cohort (Heritability = 0.24, *p* = 0.011). We identified several GWS SNPs for EAs at the *LDHB* locus; and *LDHB* was also identified as GWS in the gene-based association analysis. The SNPs identified in and around this gene were replicated in an independent cohort of LATs (rs1677091, *p* = 6.5 × 10^−3^, EA-LAT meta *p* = 4.7 × 10^−9^) but not EAs (*p* = 0.74, EA-EA meta *p* = 0.02). This ubiquitously-expressed gene encodes the B subunit of the lactate dehydrogenase enzyme and is vital in the anaerobic conversion of NADH to NAD + and lactate to pyruvate and conversion between NAD + and NADH. Increases in expression of LDHB protein have been found in peripheral blood mononuclear cells of first onset antipsychotic naive schizophrenics^34^.

Gene-based association analysis of data from EAs also identified *PHLDB2*. Previous studies have found increased expression of this gene in peripheral blood from subjects with increasing suicidal thoughts and behaviors when followed longitudinally^35,36^, The increase occurred when the subject’s suicidal thoughts and behaviors increased. *PHLDB2* may warrant further study as a possible peripheral blood biomarker candidate for suicidal risk.

The third gene identified for EAs with gene-based association analysis was *PGBD5*; it maps 5 MB from “Nucleoporin 133” (*NUP133*); the latter locus was epigenome-wide significant in a study using a quantitative suicidality score^37^. Although the authors of that study focused more on *NUP133*, *PGBD5* was discussed for its proximity to top findings and overlap with findings in panic-induced epigenetic H3K27me3 modifications in mice^38^. Recent panic attacks and the diagnosis of panic disorder within 12-months are significantly related to suicidal ideation and attempts, even when co-morbidities are controlled for^39^. We speculate that the role of this gene in acute panic may be relevant to suicide attempts.

Overall, SNP-based heritability within AAs was ascertained by GCTA on unrelated subjects in the AA cohort (Heritability = 0.13, p = 0.026). Several GWS loci for the severity of suicide attempts were identified in AAs. The lead SNP (rs683813) maps close to *ARNTL2-AS1*. *ARNTL2*, also known as *BMAL2*, is a paralog of *BMAL1* and plays an important role in circadian rhythms and regulation. SNPs in *ARNTL2* have shown suggestive evidence for association with rapid cycling of mood along with a diurnal phenotype of worsening mood in the evening^40^. A candidate gene study identified a polymorphism in *ARNTL2* that was nominally related to morning preference in 966 British participants^41^. Suicide biomarker studies in the peripheral blood of U.S. participants have highlighted genes whose expression tracks longitudinal changes in suicidal ideation and may be enriched for core regulators of the circadian clock^33,35^. This observation suggests further that time-of-day and season-of-the-year may be noteworthy factors that affect suicide biology. Additional studies will be needed to understand this mechanism potentially associated with suicidal behaviors.

The lead GWS SNP (rs72740082) on chromosome 15 maps to the *CTXND1*, *LINC01314 and FAH* locus. The same SNP was also GWS when run in the Yale-Penn trans-population meta-analysis of EAs and AAs. This SNP was not directly available in the STARRS sample replication, but an adjacent SNP in high LD with it that was nearly-GWS in the Yale-Penn meta-analysis (rs72740088, *D*’ = 0.95, *R*^2^ = 0.68, *p* = 7.49 × 10^−8^) was nominally significant in the STARRS AA sample (*p* = 5.22 × 10^−3^). When the STARRS AA and Yale-Penn samples were meta-analyzed together the *p* value improved (*p* = 1.51 × 10^−9^). An overall meta of all Yale-Penn and STARRS summary statistics yields an overall *p* = 7.84 × 10^−7^. In many of these situations, lack of availability of the actual Yale-Penn lead SNPs in other samples likely diminished the statistical significance of the final meta-analyzed results. Overexpression of *LINC01314* was recently shown to act as a tumor suppressor in hepatoblastoma, possibly through the Hippo-YAP signaling pathway^42^. This pathway has been previously implicated in psychiatric conditions such as schizophrenia and anxiety through one of its key effectors, the “Nitric Oxide Synthase 1 Adaptor Protein” (*NOS1AP)*. Interestingly, rs72740082 is in LD with another SNP (rs75782446, *D*’ = 0.79, *R*^2^ = 0.44) found within an intron of the *FAH* gene. This gene acts as the last enzyme in the catabolic pathway which breaks down the amino acid tyrosine, used for synthesis of dopamine and the other catecholamine neurotransmitters.

African ancestry is underrepresented in the literature. This is the largest GWAS of suicide to date in African Americans and we identified several GWS signals in AAs. Although these were with moderately-low MAF SNPs (0.03 range), there was no evidence of genomic inflation due to potential confounders. To evaluate whether the effects were biased by the distribution of our quantitative phenotype, we ran 1,000 permutations with randomly assigned phenotypes all sharing the same distribution of the primary experiment. The results (Supplementary Figure S1) failed to show that bias was introduced by the phenotype distribution.

The lead SNP for AAs on chromosome 18 maps to an intergenic region ~680 kb from the microRNA 4318 (*MIR4318*). This SNP association is a novel finding. It does not appear in the GWAS catalog nor is it a known eQTL based on lookups in the online databases BRAINEAC or GTEx. This likely reflects, in part, the sparsity of GWS findings among AAs, who are understudied, and suggests that it may identify an AA-specific functional locus.

The gene-based association identified a single gene as GWS for AAs, the “NMDA Receptor-Regulated Protein 2” *(NARG2)*. This gene is also known as the “Interactor of Little Elongation Complex ELL Subunit 2” *(ICE2)*. *NARG2 (ICE2)* is an NMDA receptor regulated protein component of the little elongation complex (LEC), which plays a role in small nuclear RNA (snRNA) transcription^43,44^.

PRS identified significant genetic overlap between MDD and suicide attempt severity; the PGC-MDD PRS explains up to 0.7% of the variance of suicide attempt severity in the Yale-Penn cohort. MDD is a well-known risk factor for suicidal behaviors^45–47^, with significantly increased attempts and death by suicide when compared to the general population.

We focused on suicide attempts by excluding individuals with suicidal ideation, which is likely to be significantly more heterogeneous; this reduced unaccounted for and potentially confounding complexity in the phenotype. Removing suicidal ideation also reduced the concern that individuals with a related and sometimes overlapping phenotype, that may not be on the same spectrum in every individual, would be misclassified. Depending on their future action, subjects in the “ideation” category could eventually attempt suicide (which they would be expected to do at a higher rate than subjects without ideation).

MAGMA gene-set analysis identified the caspace pathway Dwivedi et al.^48^ previously identified an increased ratio of p75^NTR^ to Trk receptors, which has been shown to be pro-apoptotic, in the brains of suicide completers. Pathway analysis of biomarkers for suicidal behavior has also identified apoptosis as an enriched biological pathway for suicide^49^. This could be related to stress or other interacting co-morbidities and may warrant further study as an intervention or risk-detection mechanism.

Our study had several limitations. Our cohort is reasonably large compared to other published GWAS of suicidal behaviors, but larger-yet studies are needed to confirm these results and to identify low effect size genetic risk factors. Additionally, the EA findings replicated only in the LAT sample, and not the EA sample in the STARRS cohort. This curious result could reflect the inclusion of Latino individuals in the Yale-Penn EA cohort or reflect the fact that that the participants in the Army-STARRS sample were much younger and had traversed less of their lifetime risk period for suicide. Accordingly, the Army-STARRS suicide behavior had earlier age of onset; for many traits this corresponds with greater severity. It would be highly interesting to follow these individuals longitudinally to evaluate the evolutions of this phenotype.

We also considered the possibility that, because the Yale-Penn sample is made up of subjects recruited for SUD studies (affecteds and controls), our results may be more specific to suicide attempt severity in SUD. We looked up our top findings for suicide in previous published GWAS for alcohol, cocaine, and cannabis dependence performed in the same cohort but found no evidence for even nominal association with SUDs.

In summary, we identified several associations in genes or associated pathways that have previously been implicated in suicide. Epigenetic modifications near *PGBD5* and *NUP133* have been associated with quantitative scores for suicidality^37^. Suicidal ideation state changes have been associated with the increased expression of *PHLDB2* in independent studies in men^36^ and women^35^. We also identified several loci that are novel for suicide but have previously been associated with psychiatric disorders that overlap with risk for suicidal behavior^50^. Increased *LDHB* expression has been associated with the first onset of psychosis in antipsychotic-naïve schizophrenia patients^34^. *ARNTL2* and other core regulators of circadian rhythms have frequently been associated with bipolar disorder. Individuals with these disorders are at elevated risk of suicidal behavior. In addition to the novel findings, the results highlight convergent evidence in the literature of molecular genetic components as risk factors for suicidal behavior and predicted pleiotropy between psychiatric disorders and suicide attempts.

Suicidal behavior is heterogeneous and is driven by many complex interacting environmental and genetic influences. It is unlikely that any single gene could exert a large enough effect to drive such complexity (or capture the environmental influence), but although single variants may contribute only small effects individually, considered together, they may collectively provide new insight into the genetic risk factors underlying suicidal behaviors. Our success at identifying significant associations even in a comparatively modest-sized sample, bodes well for the future of this endeavor.

## Supplementary information

Supplemental Information
