# Supplementary material for: Genetic associations with suicide attempt severity and genetic overlap with major depression
Source: Transl Psychiatry. 2019 Jan 17;9:22. doi: 10.1038/s41398-018-0340-2 (PMC6336846; doi:10.1038/s41398-018-0340-2)
Supplement: Supplementary file 1 — Supplemental Information [file 41398_2018_340_MOESM1_ESM.docx]

**Supplementary Material**


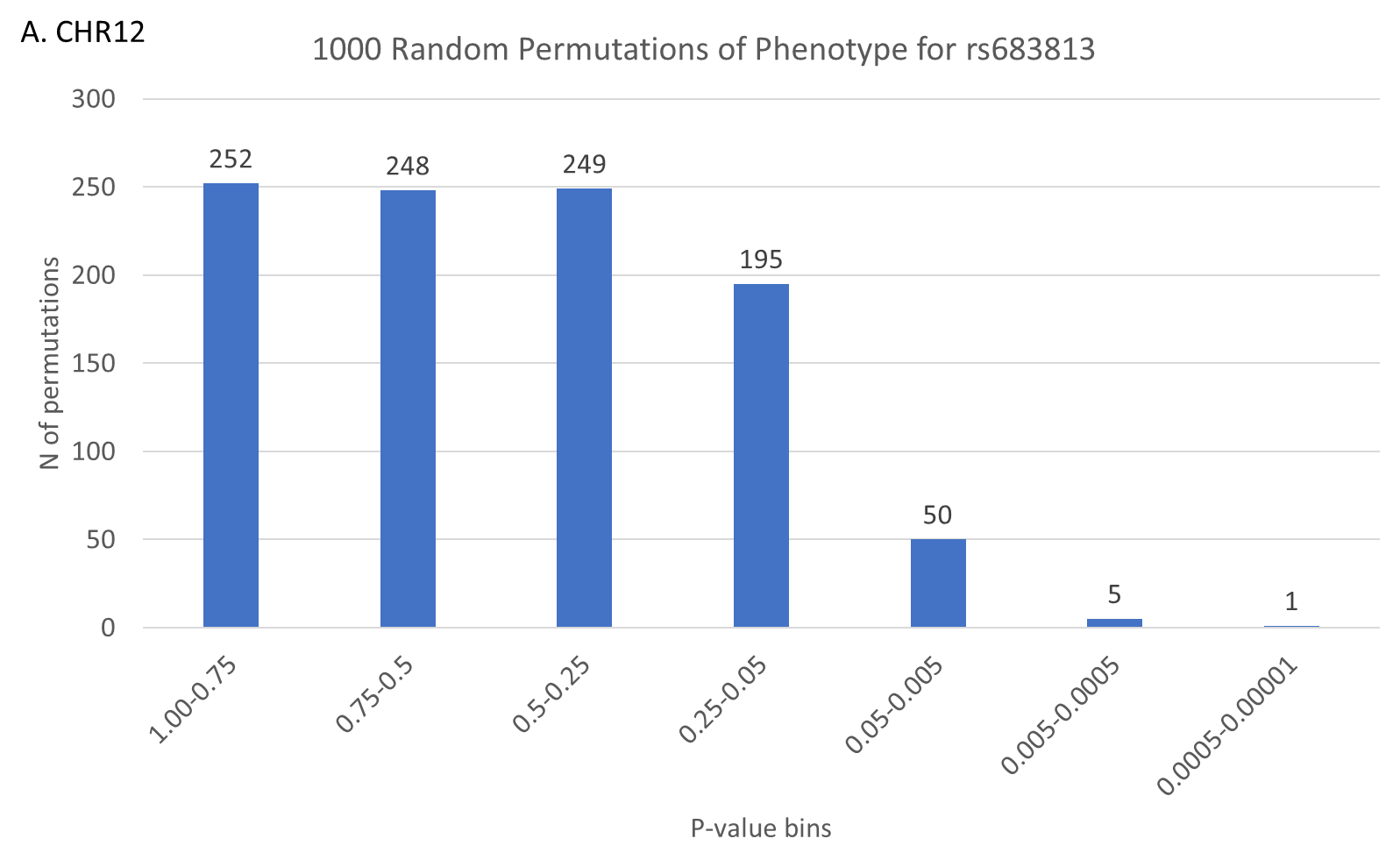


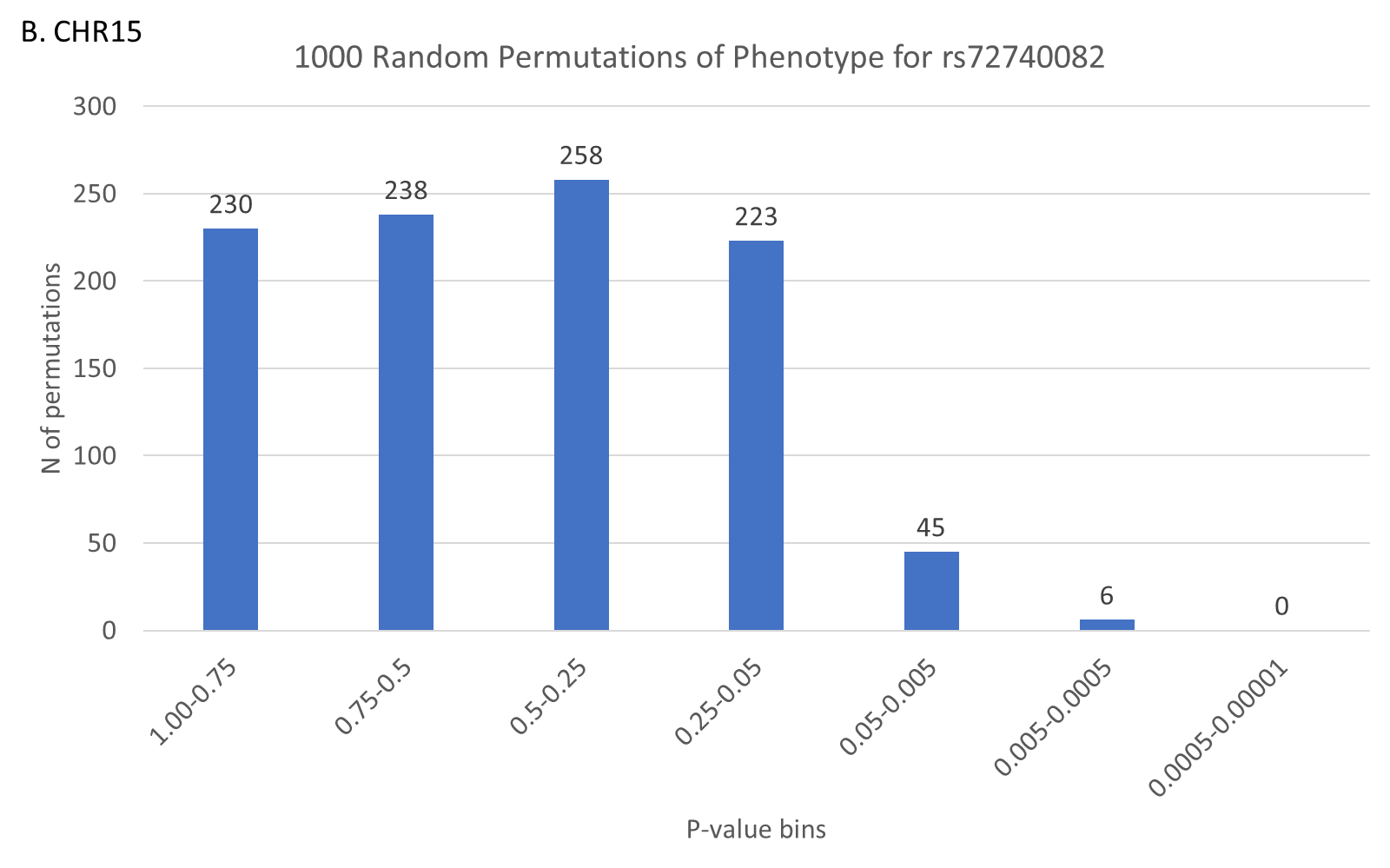


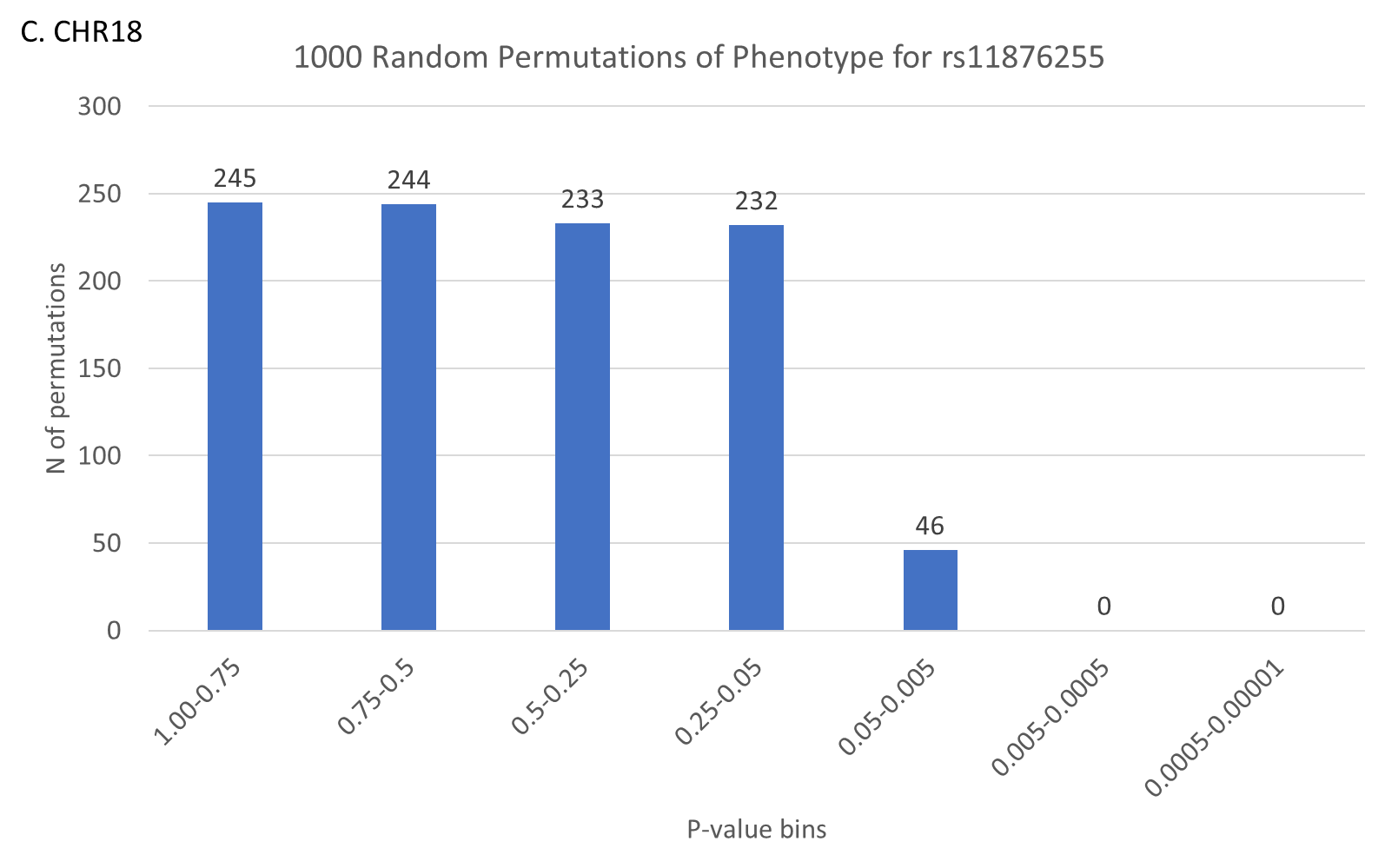


Supplementary Figure S1. Random Permutations of Phenotype Distribution Produce no More Significant Hits Than Expected By Chance.


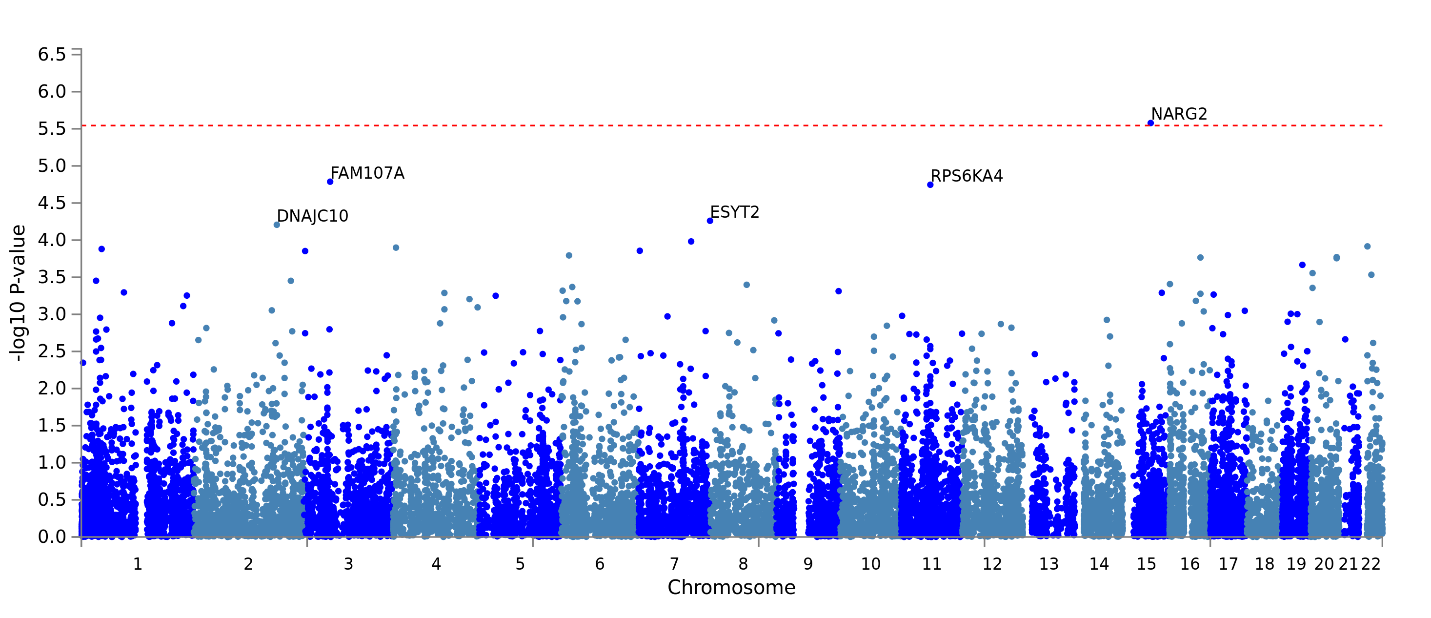


Supplementary Figure 2. FUMA Gene-Based Analysis of AAs Suicide Attempt Severity

European Americans Suicide Attempt Severity


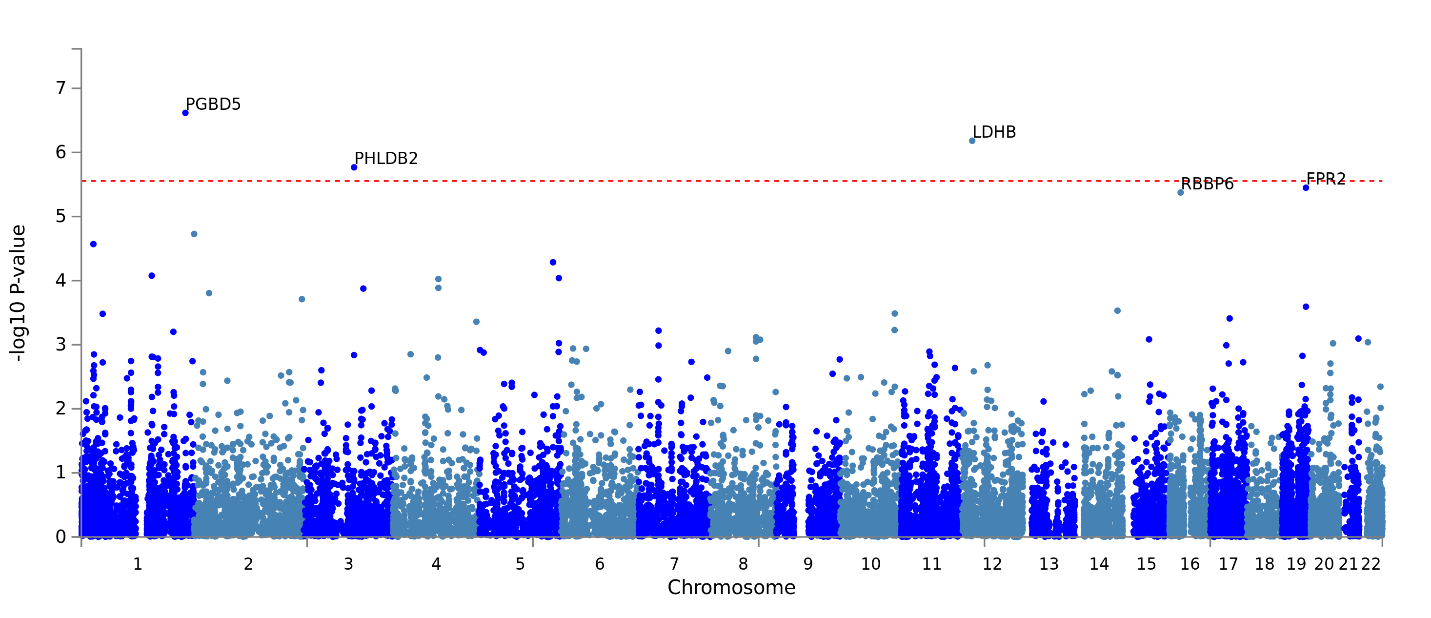


Supplementary Figure S3. FUMA Gene-Based Analysis of EAs Suicide Attempt Severity

Sensitivity Analyses:

EA

Supplementary Figure S4. Affected allele frequency at each level of the scale in EAs.

AA

Supplementary Figure S5. Affected allele frequency at each level of the scale in AAs.

|  | Subjects | | | | |
| --- | --- | --- | --- | --- | --- |
|  | AAYP1 | AAYP2 | EAYP1 | EAYP2 | Total |
| Total Subjects with Complete Phenotype and Genotype Data | 3191 | 1707 | 1725 | 1696 | 8318 |
| Final Cohort with Suicidal Ideation Removed | 2530 | 1351 | 1225 | 1214 | 6320 |
| % Excluded due to Ideation without Attempt | 20.7% | 20.9% | 29.0% | 28.4% | 24.0% |

Supplementary Table S1. Breakdown of the Yale-Penn total sample accounting for number of excluded individuals with suicidal ideation.

## Supplementary Table S2. Top findings in meta-analysis of Yale-Penn EA Attempts.

| Marker Name | rsid | BP | Allele1 | Allele2 | n | Zscore | P.value | Direction |
| --- | --- | --- | --- | --- | --- | --- | --- | --- |
| CHR12:21780851 | **rs1677091** | **21780851** | **a** | **c** | **2439** | **5.719** | **1.07E-08** | **++** |
| CHR12:21810646 | **rs11046147** | **21810646** | **t** | **c** | **2439** | **5.717** | **1.09E-08** | **++** |
| CHR12:21774268 | **rs255447** | **21774268** | **t** | **c** | **2439** | **-5.69** | **1.27E-08** | **--** |
| CHR12:21815979 | **rs12825320** | **21815979** | **c** | **g** | **2439** | **5.641** | **1.69E-08** | **++** |
| CHR12:21784608 | **rs4762855** | **21784608** | **t** | **c** | **2439** | **5.614** | **1.97E-08** | **++** |
| CHR12:21816195 | **rs7302534** | **21816195** | **a** | **g** | **2439** | **-5.576** | **2.47E-08** | **--** |
| CHR12:21815425 | **rs56125867** | **21815425** | **t** | **c** | **2439** | **-5.552** | **2.83E-08** | **--** |
| CHR12:21784041 | **rs10841869** | **21784041** | **t** | **c** | **2439** | **-5.551** | **2.84E-08** | **--** |
| CHR12:21816563 | **rs7303185** | **21816563** | **a** | **c** | **2439** | **5.541** | **3.01E-08** | **++** |
| CHR12:21787008 | **rs1677137** | **21787008** | **a** | **t** | **2439** | **-5.535** | **3.11E-08** | **--** |
| CHR12:21816831 | **rs7303612** | **21816831** | **t** | **c** | **2439** | **5.531** | **3.18E-08** | **++** |
| CHR12:21817453 | **rs58134911** | **21817453** | **a** | **t** | **2439** | **5.531** | **3.18E-08** | **++** |
| CHR12:21817542 | **rs11046156** | **21817542** | **t** | **c** | **2439** | **-5.531** | **3.18E-08** | **--** |
| CHR12:21816004 | **rs12824993** | **21816004** | **t** | **c** | **2439** | **5.521** | **3.37E-08** | **++** |
| CHR12:21816005 | **rs12824994** | **21816005** | **a** | **c** | **2439** | **5.521** | **3.37E-08** | **++** |
| CHR12:21779355 | **rs10841862** | **21779355** | **t** | **c** | **2439** | **-5.497** | **3.86E-08** | **--** |
| CHR12:21813860 | **rs34438801** | **21813860** | **g** | **ga** | **2439** | **5.488** | **4.07E-08** | **++** |
| CHR12:21818102 | **rs34313697** | **21818102** | **g** | **ga** | **2439** | **-5.481** | **4.24E-08** | **--** |
| CHR12:21818267 | **rs7297042** | **21818267** | **a** | **t** | **2439** | **5.473** | **4.41E-08** | **++** |
| CHR12:21814496 | **rs10841873** | **21814496** | **a** | **t** | **2439** | **-5.472** | **4.45E-08** | **--** |
| CHR12:21814529 | **rs11836830** | **21814529** | **a** | **c** | **2439** | **-5.472** | **4.45E-08** | **--** |
| CHR12:21814566 | **rs11832180** | **21814566** | **a** | **c** | **2439** | **5.472** | **4.45E-08** | **++** |
| CHR12:21814568 | **rs11832211** | **21814568** | **t** | **g** | **2439** | **5.472** | **4.45E-08** | **++** |
| CHR12:21814574 | **rs11834600** | **21814574** | **t** | **c** | **2439** | **-5.472** | **4.45E-08** | **--** |
| CHR12:21815564 | **rs74184609** | **21815564** | **t** | **c** | **2439** | **-5.465** | **4.62E-08** | **--** |
| CHR12:21815580 | **rs73077300** | **21815580** | **t** | **c** | **2439** | **-5.465** | **4.62E-08** | **--** |
| CHR12:21815612 | **rs78929102** | **21815612** | **c** | **g** | **2439** | **5.465** | **4.62E-08** | **++** |
| CHR12:21815650 | **rs76654229** | **21815650** | **a** | **c** | **2439** | **-5.465** | **4.62E-08** | **--** |
| CHR12:21815665 | **rs80042173** | **21815665** | **a** | **c** | **2439** | **-5.465** | **4.62E-08** | **--** |
| CHR12:21815725 | **rs66978947** | **21815725** | **a** | **g** | **2439** | **5.465** | **4.62E-08** | **++** |
| CHR12:21813877 | **rs7135965** | **21813877** | **a** | **g** | **2439** | **5.465** | **4.64E-08** | **++** |
| CHR12:21816351 | **rs7302889** | **21816351** | **t** | **c** | **2439** | **5.457** | **4.83E-08** | **++** |
| CHR12:21816357 | **rs7133707** | **21816357** | **t** | **c** | **2439** | **-5.457** | **4.83E-08** | **--** |
| CHR12:21815874 | **rs72276276** | **21815874** | **ccccactt** | **c** | **2439** | **-5.456** | **4.88E-08** | **--** |
| CHR12:21814682 | rs11046148 | 21814682 | a | g | 2439 | -5.451 | 5.02E-08 | -- |
| CHR12:21814745 | rs10841874 | 21814745 | t | c | 2439 | -5.451 | 5.02E-08 | -- |
| CHR12:21814783 | rs11046149 | 21814783 | c | g | 2439 | -5.451 | 5.02E-08 | -- |
| CHR12:21814852 | rs11046150 | 21814852 | a | c | 2439 | 5.451 | 5.02E-08 | ++ |
| CHR12:21814908 | rs11046151 | 21814908 | a | g | 2439 | -5.451 | 5.02E-08 | -- |
| CHR12:21815104 | rs12824571 | 21815104 | t | c | 2439 | -5.451 | 5.02E-08 | -- |
| CHR12:21815232 | rs12823853 | 21815232 | c | g | 2439 | 5.451 | 5.02E-08 | ++ |
| CHR12:21815343 | rs67247140 | 21815343 | t | c | 2439 | -5.451 | 5.02E-08 | -- |
| CHR12:21815668 | rs146857244 | 21815668 | a | ac | 2439 | 5.451 | 5.02E-08 | ++ |
| CHR12:21815800 | rs12826014 | 21815800 | t | c | 2439 | -5.451 | 5.02E-08 | -- |
| CHR12:21815820 | rs12824936 | 21815820 | c | g | 2439 | 5.451 | 5.02E-08 | ++ |
| CHR12:21815898 | rs12824788 | 21815898 | a | c | 2439 | 5.451 | 5.02E-08 | ++ |
| CHR12:21816031 | rs12829701 | 21816031 | a | c | 2439 | -5.451 | 5.02E-08 | -- |
| CHR12:21816082 | rs12830306 | 21816082 | c | g | 2439 | 5.451 | 5.02E-08 | ++ |
| CHR12:21816205 | rs7302639 | 21816205 | t | c | 2439 | 5.451 | 5.02E-08 | ++ |
| CHR12:21816889 | rs7303644 | 21816889 | c | g | 2439 | -5.451 | 5.02E-08 | -- |
| CHR12:21816920 | rs7303528 | 21816920 | a | t | 2439 | -5.451 | 5.02E-08 | -- |
| CHR12:21817003 | rs11046153 | 21817003 | a | g | 2439 | 5.451 | 5.02E-08 | ++ |
| CHR12:21817261 | rs11046154 | 21817261 | a | g | 2439 | 5.451 | 5.02E-08 | ++ |
| CHR12:21816349 | rs7133704 | 21816349 | t | c | 2439 | -5.448 | 5.09E-08 | -- |
| CHR12:21816335 | rs7303126 | 21816335 | a | g | 2439 | 5.432 | 5.57E-08 | ++ |
| CHR12:21815421 | rs71436855 | 21815421 | a | g | 2439 | -5.43 | 5.65E-08 | -- |
| CHR12:21815427 | rs71436857 | 21815427 | a | g | 2439 | -5.43 | 5.65E-08 | -- |
| CHR12:21815440 | rs79594593 | 21815440 | a | c | 2439 | -5.43 | 5.65E-08 | -- |
| CHR12:21815389 | rs67286733 | 21815389 | a | c | 2439 | 5.426 | 5.76E-08 | ++ |
| CHR12:21778904 | rs10841860 | 21778904 | a | g | 2439 | 5.419 | 5.98E-08 | ++ |
| CHR12:21814162 | rs10841872 | 21814162 | c | g | 2439 | 5.414 | 6.17E-08 | ++ |
| CHR12:21815488 | rs74184947 | 21815488 | c | g | 2439 | 5.405 | 6.48E-08 | ++ |
| CHR12:21815491 | rs74207710 | 21815491 | a | t | 2439 | 5.405 | 6.48E-08 | ++ |
| CHR12:21815504 | rs74219429 | 21815504 | t | c | 2439 | -5.405 | 6.48E-08 | -- |
| CHR12:21760286 | rs34600045 | 21760286 | t | c | 2439 | 5.327 | 1.00E-07 | ++ |
| CHR12:21781246 | rs10841868 | 21781246 | t | g | 2439 | 5.276 | 1.32E-07 | ++ |
| CHR12:21807687 | rs7953579 | 21807687 | c | g | 2439 | 5.271 | 1.36E-07 | ++ |
| CHR12:21781181 | rs10841867 | 21781181 | a | g | 2439 | -5.262 | 1.43E-07 | -- |
| CHR12:21816621 | rs368907914 | 21816621 | caaaaaaaa | c | 2439 | -5.251 | 1.51E-07 | -- |
| CHR12:21825201 | rs7133519 | 21825201 | t | g | 2439 | 5.234 | 1.66E-07 | ++ |
| CHR12:21826222 | rs34031159 | 21826222 | a | g | 2439 | -5.234 | 1.66E-07 | -- |
| CHR12:21819568 | rs11046161 | 21819568 | a | t | 2439 | 5.211 | 1.88E-07 | ++ |
| CHR12:21819404 | rs11046159 | 21819404 | c | g | 2439 | 5.203 | 1.96E-07 | ++ |
| CHR12:21842821 | rs7975931 | 21842821 | t | g | 2439 | -5.168 | 2.36E-07 | -- |
| CHR12:21819535 | rs11046160 | 21819535 | t | c | 2439 | -5.163 | 2.43E-07 | -- |
| CHR12:21819641 | rs7301852 | 21819641 | t | c | 2439 | -5.163 | 2.43E-07 | -- |
| CHR12:21819667 | rs7316427 | 21819667 | a | g | 2439 | 5.163 | 2.43E-07 | ++ |
| CHR12:21819702 | rs7301883 | 21819702 | a | t | 2439 | 5.163 | 2.43E-07 | ++ |
| CHR12:21819842 | rs7316669 | 21819842 | a | g | 2439 | 5.163 | 2.43E-07 | ++ |
| CHR12:21819903 | rs7316475 | 21819903 | t | c | 2439 | 5.163 | 2.43E-07 | ++ |
| CHR12:21820070 | rs7305475 | 21820070 | t | c | 2439 | -5.163 | 2.43E-07 | -- |
| CHR12:21803685 | rs1677084 | 21803685 | c | g | 2439 | -5.157 | 2.50E-07 | -- |
| CHR12:21803770 | rs1650307 | 21803770 | t | c | 2439 | -5.157 | 2.50E-07 | -- |
| CHR12:21820146 | rs10686695 | 21820146 | cagag | c | 2439 | 5.154 | 2.55E-07 | ++ |
| CHR12:21838885 | rs34084105 | 21838885 | a | g | 2439 | 5.154 | 2.55E-07 | ++ |
| CHR12:21839397 | rs10841878 | 21839397 | c | g | 2439 | -5.154 | 2.55E-07 | -- |
| CHR12:21827614 | rs12426826 | 21827614 | t | c | 2439 | 5.148 | 2.64E-07 | ++ |
| CHR12:21820578 | rs17629491 | 21820578 | a | g | 2439 | -5.142 | 2.72E-07 | -- |
| CHR12:21831069 | rs12426310 | 21831069 | t | g | 2439 | 5.142 | 2.72E-07 | ++ |
| CHR12:21834560 | rs66592830 | 21834560 | a | c | 2439 | 5.142 | 2.72E-07 | ++ |
| CHR12:21834822 | rs34015001 | 21834822 | a | c | 2439 | 5.142 | 2.72E-07 | ++ |
| CHR12:21826254 | rs34822889 | 21826254 | c | g | 2439 | 5.137 | 2.79E-07 | ++ |
| CHR12:21823036 | rs10770848 | 21823036 | a | g | 2439 | 5.127 | 2.94E-07 | ++ |
| CHR12:21839933 | rs2077105 | 21839933 | a | g | 2439 | -5.127 | 2.94E-07 | -- |
| CHR12:21819125 | rs11046157 | 21819125 | t | c | 2439 | 5.123 | 3.00E-07 | ++ |
| CHR12:21815235 | rs12823507 | 21815235 | c | g | 2439 | -5.12 | 3.06E-07 | -- |
| CHR12:21817385 | rs187647634 | 21817385 | c | g | 2439 | -5.111 | 3.21E-07 | -- |
| CHR12:21819137 | rs11046158 | 21819137 | a | t | 2439 | 5.109 | 3.24E-07 | ++ |
| CHR12:21815454 | rs201502983 | 21815454 | a | aagag | 2439 | 5.101 | 3.38E-07 | ++ |
| CHR12:21815463 | rs143849831 | 21815463 | a | t | 2439 | -5.101 | 3.38E-07 | -- |
| CHR12:21815467 | rs148611933 | 21815467 | t | c | 2439 | 5.101 | 3.38E-07 | ++ |
| CHR12:21815472 | rs77765937 | 21815472 | c | g | 2439 | -5.101 | 3.38E-07 | -- |
| CHR12:21843576 | rs11046164 | 21843576 | t | c | 2439 | 5.083 | 3.72E-07 | ++ |
| CHR12:21845290 | rs10770851 | 21845290 | c | g | 2439 | 5.077 | 3.84E-07 | ++ |
| CHR12:21841978 | rs10841880 | 21841978 | a | t | 2439 | -5.063 | 4.13E-07 | -- |
| CHR12:21849348 | rs852816 | 21849348 | t | c | 2439 | -5.062 | 4.15E-07 | -- |
| CHR12:21850983 | rs852817 | 21850983 | t | c | 2439 | -5.045 | 4.53E-07 | -- |
| CHR12:21858180 | rs860447 | 21858180 | t | c | 2439 | 4.988 | 6.10E-07 | ++ |
| CHR12:21853012 | rs11046166 | 21853012 | a | g | 2439 | 4.986 | 6.16E-07 | ++ |
| CHR16:24558653 | rs9927702 | 24558653 | a | g | 2439 | -4.953 | 7.32E-07 | -- |
| CHR12:21857409 | rs11046170 | 21857409 | t | g | 2439 | 4.943 | 7.70E-07 | ++ |
| CHR10:71788611 | rs111394656 | 71788611 | t | c | 2439 | 4.938 | 7.91E-07 | ++ |
| CHR16:24570381 | rs7200560 | 24570381 | a | t | 2439 | 4.928 | 8.30E-07 | ++ |

Supplementary Table S3. Top findings in meta-analysis of Yale-Penn AA Attempts.

| Marker Name | rsid | BP | Allele1 | Allele2 | n | Zscore | P.value | Direction |
| --- | --- | --- | --- | --- | --- | --- | --- | --- |
| CHR12:27600331 | **rs683813** | **27600331** | **t** | **c** | **3881** | **-5.606** | **2.07E-08** | **--** |
| CHR15:80508139 | **rs72740082** | **80508139** | **a** | **t** | **3881** | **-5.583** | **2.36E-08** | **--** |
| CHR18:35911354 | **rs11876255** | **35911354** | **a** | **g** | **3881** | **-5.466** | **4.61E-08** | **--** |
| CHR15:80512515 | rs72740088 | 80512515 | t | c | 3881 | -5.379 | 7.49E-08 | -- |
| CHR3:158144979 | rs61537580 | 1.58E+08 | a | ac | 3881 | 5.233 | 1.67E-07 | ++ |
| CHR3:157675069 | rs9290009 | 1.58E+08 | a | g | 3881 | 5.221 | 1.78E-07 | ++ |
| CHR3:185622421 | rs6795254 | 1.86E+08 | t | c | 3881 | -5.218 | 1.81E-07 | -- |
| CHR18:35912717 | rs7240979 | 35912717 | t | c | 3881 | -5.171 | 2.33E-07 | -- |
| CHR11:96032273 | rs76182109 | 96032273 | a | g | 3881 | -5.167 | 2.38E-07 | -- |
| CHR13:50967576 | rs113498843 | 50967576 | a | g | 3881 | 5.123 | 3.01E-07 | ++ |
| CHR13:51015161 | rs56155205 | 51015161 | c | g | 3881 | 5.112 | 3.19E-07 | ++ |
| CHR13:51020490 | rs75389317 | 51020490 | a | t | 3881 | -5.112 | 3.19E-07 | -- |
| CHR18:35954120 | rs8092042 | 35954120 | t | c | 3881 | 5.105 | 3.32E-07 | ++ |
| CHR18:35955209 | rs144723193 | 35955209 | a | c | 3881 | -5.105 | 3.32E-07 | -- |
| CHR2:173351176 | rs16860543 | 1.73E+08 | a | g | 3881 | 5.048 | 4.48E-07 | ++ |
| CHR2:173355907 | rs56084828 | 1.73E+08 | a | c | 3881 | -5.048 | 4.48E-07 | -- |
| CHR9:83116342 | rs12683724 | 83116342 | a | g | 3881 | -5.037 | 4.73E-07 | -- |
| CHR9:83117229 | rs10867557 | 83117229 | a | t | 3881 | -5.037 | 4.73E-07 | -- |
| CHR18:35960620 | rs151078736 | 35960620 | t | c | 3881 | -5.035 | 4.78E-07 | -- |
| CHR18:35961612 | rs75346662 | 35961612 | t | g | 3881 | 5.008 | 5.51E-07 | ++ |
| CHR18:35961928 | rs75158723 | 35961928 | t | g | 3881 | -5.008 | 5.51E-07 | -- |
| CHR18:35962020 | rs77672600 | 35962020 | a | g | 3881 | 5.008 | 5.51E-07 | ++ |
| CHR13:51007824 | rs76886430 | 51007824 | t | c | 3881 | -4.976 | 6.49E-07 | -- |
| CHR18:35962676 | rs11873140 | 35962676 | a | g | 3881 | -4.975 | 6.54E-07 | -- |
| CHR18:35967146 | rs150919601 | 35967146 | t | c | 3881 | 4.953 | 7.30E-07 | ++ |
| CHR5:35868562 | rs11567739 | 35868562 | a | c | 3881 | 4.949 | 7.45E-07 | ++ |
| CHR5:35869044 | rs9282748 | 35869044 | t | c | 3881 | 4.949 | 7.45E-07 | ++ |
| CHR1:214879192 | rs61520094 | 2.15E+08 | t | c | 3881 | 4.932 | 8.14E-07 | ++ |
| CHR9:105349079 | rs76477759 | 1.05E+08 | c | g | 3881 | -4.923 | 8.53E-07 | -- |
| CHR13:50988880 | rs55810037 | 50988880 | t | c | 3881 | -4.901 | 9.51E-07 | -- |

Supplementary Table S4. Army-STARRS phenotype

| Cohort | Total Participants | Army-STARRS Participants with severity score | | |
| --- | --- | --- | --- | --- |
|  |  | 0 | 1 | 2 |
|  |  | No Suicidal Ideation/thoughts | Suicide Attempt | Violent Suicide Attempt |
| AFR | 1488 | 1405 | 48 | 35 |
| EUR | 9382 | 8977 | 230 | 175 |
| LAT | 2963 | 2854 | 66 | 43 |

Supplementary Table S5. Age and sex in the discovery (Yale-Penn) and replication (Army-STARRS) cohorts.

| Cohort | % Female | Age(Mean(SD)) |
| --- | --- | --- |
| EA Yale-Penn 1 | 41.4% | 37.907(10.901) |
| EA Yale-Penn 2 | 41.6% | 39.168(12.811) |
| EA Army-STARRS NSS1 | 14.1% | 21.117(3.375) |
| EA Army-STARRS NSS2 | 19.0% | 20.311(3.273) |
| EA Army-STARRS PPDS | 5.3% | 25.796(5.858) |
| LAT Army-STARRS NSS1 | 17.6% | 21.035(3.266) |
| LAT Army-STARRS NSS2 | 24.8% | 20.103(2.983) |
| LAT Army-STARRS PPDS | 8.2% | 25.171(5.566) |
| AA Yale-Penn 1 | 48.5% | 41.126(9.068) |
| AA Yale-Penn 2 | 42.3% | 40.89(11.028) |
| AA Army-STARRS NSS1 | 30.2% | 21.045(3.156) |
| AA Army-STARRS NSS2 | 36.0% | 20.628(3.159) |

Supplementary Table S6. Comorbidity in the Yale-Penn cohort

Trans-population Meta-analysis

Two independent GWS associations were identified in the trans-population analysis of the Yale-Penn sample, with six GWS SNPs. The top signal was found on chromosome 5 (rs201402387, MAF=0.04, p=1.54×10^-8^) ~8kb from a small noncoding RNA pseudogene. This SNP was nominally significant in both AAs and EAs (AA p= 3.12×10^-6^, EA p= 1.26×10^-3^) with MAF ranging between 0.03 and 0.04. The other signal was near *CTXND1* and *LINC01314*, with a similar association strength (rs72740082, MAF=0.06, p=2.43×10^-8^). This SNP is the same that was identified GWS in Yale-Penn AAs (MAF=0.03, p=2.36×10^-8^) and was nearly nominally significant in EAs with the same effect direction (MAF=0.08, p=0.053).
